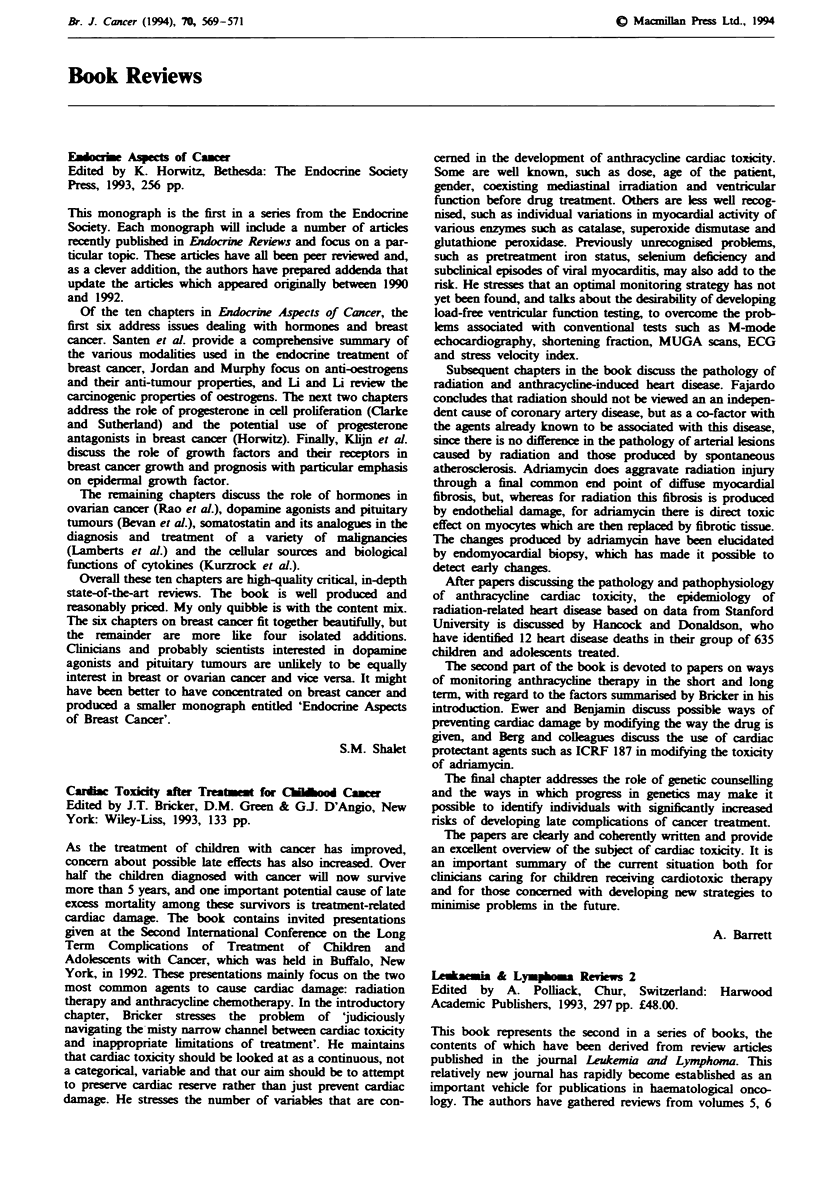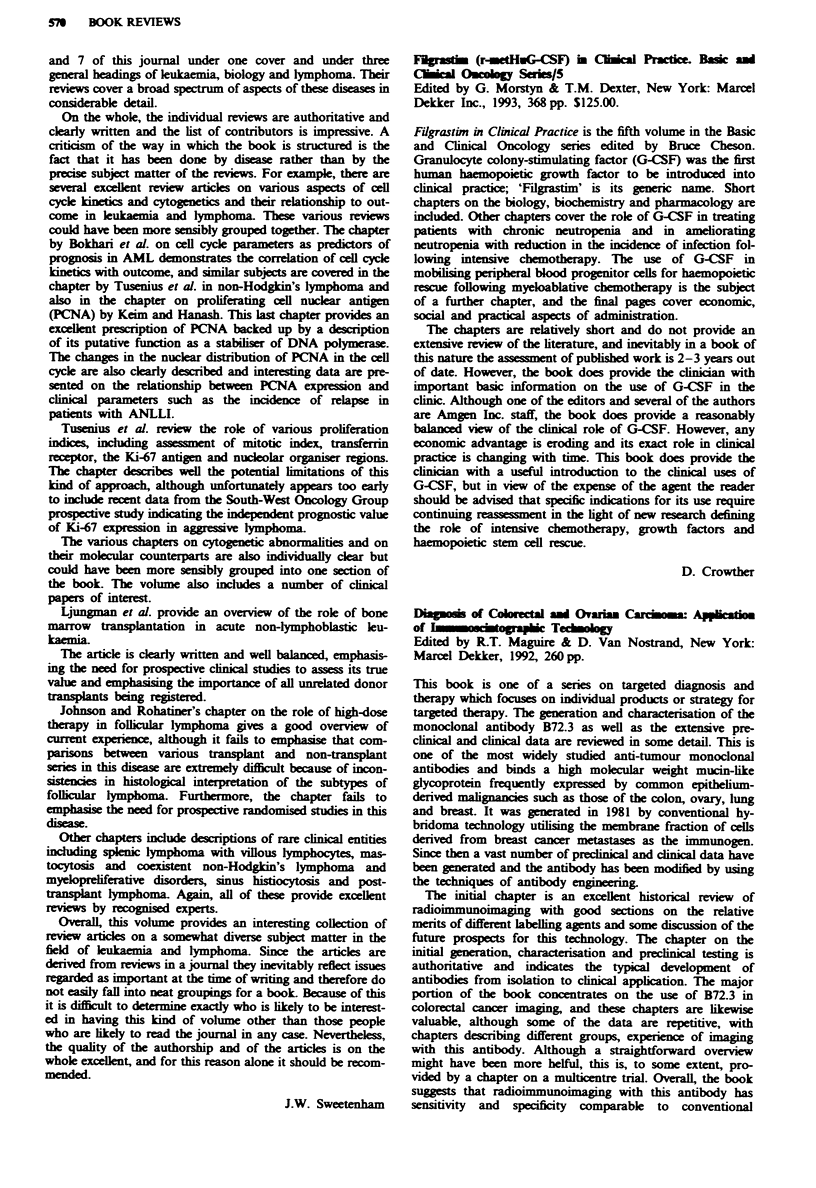# Leukaemia & lymphoma reviews 2

**Published:** 1994-09

**Authors:** J.W. Sweetenham


**Leukaemia & Lymphoma Reviews 2**
Edited by A. Polliack, Chur, Switzerland: Harwood Academic Publishers, 1993, 297 pp. £48.00.

This book represents the second in a series of books, the contents of which have been derived from review articles published in the journal *Leukemia and Lymphoma*. This relatively new journal has rapidly become established as an important vehicle for publications in haematological oncology. The authors have gathered reviews from volumes 5, 6

and 7 of this journal under one cover and under three general headings of leukaemia, biology and lymphoma. Their reviews cover a broad spectrum of aspects of these diseases in considerable detail.

On the whole, the individual reviews are authoritative and clearly written and the list of contributors is impressive. A criticism of the way in which the book is structured is the fact that it has been done by disease rather than by the precise subject matter of the reviews. For example, there are several excellent review articles on various aspects of cell cycle kinetics and cytogenetics and their relationship to outcome in leukaemia and lymphoma. These various reviews could have been more sensibly grouped together. The chapter by Bokhari *et al.* on cell cycle parameters as predictors of prognosis in AML demonstrates the correlation of cell cycle kinetics with outcome, and similar subjects are covered in the chapter by Tusenius *et al.* in non-Hodgkin's lymphoma and also in the chapter on proliferating cell nuclear antigen (PCNA) by Keim and Hanash. This last chapter provides an excellent prescription of PCNA backed up by a description of its putative function as a stabiliser of DNA polymerase. The changes in the nuclear distribution of PCNA in the cell cycle are also clearly described and interesting data are presented on the relationship between PCNA expression and clinical parameters such as the incidence of relapse in patients with ANLLI.

Tusenius *et al.* review the role of various proliferation indices, including assessment of mitotic index, transferrin receptor, the Ki-67 antigen and nucleolar organiser regions. The chapter describes well the potential limitations of this kind of approach, although unfortunately appears too early to include recent data from the South-West Oncology Group prospective study indicating the independent prognostic value of Ki-67 expression in aggressive lymphoma.

The various chapters on cytogenetic abnormalities and on their molecular counterparts are also individually clear but could have been more sensibly grouped into one section of the book. The volume also includes a number of clinical papers of interest.

Ljungman *et al.* provide an overview of the role of bone marrow transplantation in acute non-lymphoblastic leukaemia.

The article is clearly written and well balanced, emphasising the need for prospective clinical studies to assess its true value and emphasising the importance of all unrelated donor transplants being registered.

Johnson and Rohatiner's chapter on the role of high-dose therapy in follicular lymphoma gives a good overview of current experience, although it fails to emphasise that comparisons between various transplant and non-transplant series in this disease are extremely difficult because of inconsistencies in histological interpretation of the subtypes of follicular lymphoma. Furthermore, the chapter fails to emphasise the need for prospective randomised studies in this disease.

Other chapters include descriptions of rare clinical entities including splenic lymphoma with villous lymphocytes, mastocytosis and coexistent non-Hodgkin's lymphoma and myelopreliferative disorders, sinus histiocytosis and post-transplant lymphoma. Again, all of these provide excellent reviews by recognised experts.

Overall, this volume provides an interesting collection of review articles on a somewhat diverse subject matter in the field of leukaemia and lymphoma. Since the articles are derived from reviews in a journal they inevitably reflect issues regarded as important at the time of writing and therefore do not easily fall into neat groupings for a book. Because of this it is difficult to determine exactly who is likely to be interested in having this kind of volume other than those people who are likely to read the journal in any case. Nevertheless, the quality of the authorship and of the articles is on the whole excellent, and for this reason alone it should be recommended.

J.W. Sweetenham